# Primary and Immortalized Cultures of Human Proximal Tubule Cells Possess Both Progenitor and Non-Progenitor Cells That Can Impact Experimental Results

**DOI:** 10.3390/jpm13040613

**Published:** 2023-03-31

**Authors:** Swojani Shrestha, Md Ehsanul Haque, Eloho Ighofose, Merrick Mcmahon, Gazal Kalyan, Rachel Guyer, Matthew Kalonick, Julia Kochanowski, Kyle Wegner, Seema Somji, Donald A. Sens, Scott H. Garrett

**Affiliations:** Department of Pathology, School of Medicine and Health Sciences, University of North Dakota, 1301 N. Columbia Road, Stop 9037, Grand Forks, ND 58202, USA; swojani.shrestha@und.edu (S.S.); mdehsanul.haque@und.edu (M.E.H.); eloho.ighofose@und.edu (E.I.); merrick.mcmahon@und.edu (M.M.); gazal.kalyan@und.edu (G.K.); rachel.guyer@und.edu (R.G.); matthew.kalonick@und.edu (M.K.); julia.kochanowski@und.edu (J.K.); kyle.wegner@und.edu (K.W.); seema.somji@und.edu (S.S.); donald.sens@und.edu (D.A.S.)

**Keywords:** CD24, PROM1, glucose, conditioned medium, RCC, cancer stem cell, kidney progenitor cells

## Abstract

Studies have reported the presence of renal proximal tubule specific progenitor cells which co-express PROM1 and CD24 markers on the cell surface. The RPTEC/TERT cell line is a telomerase-immortalized proximal tubule cell line that expresses two populations of cells, one co-expressing PROM1 and CD24 and another expressing only CD24, identical to primary cultures of human proximal tubule cells (HPT). The RPTEC/TERT cell line was used by the authors to generate two new cell lines, HRTPT co-expressing PROM1 and CD24 and HREC24T expressing only CD24. The HRTPT cell line has been shown to express properties expected of renal progenitor cells while HREC24T expresses none of these properties. The HPT cells were used in a previous study to determine the effects of elevated glucose concentrations on global gene expression. This study showed the alteration of expression of lysosomal and mTOR associated genes. In the present study, this gene set was used to determine if pure populations of cells expressing both PROM1 and CD24 had different patterns of expression than those expressing only CD24 when exposed to elevated glucose concentrations. In addition, experiments were performed to determine whether cross-talk might occur between the two cell lines based on their expression of PROM1 and CD24. It was shown that the expression of the mTOR and lysosomal genes was altered in expression between the HRTPT and HREC24T cell lines based on their PROM1 and CD24 expression. Using metallothionein (MT) expression as a marker demonstrated that both cell lines produced condition media that could alter the expression of the MT genes. It was also determined that PROM1 and CD24 co-expression was limited in renal cell carcinoma (RCC) cell lines.

## 1. Introduction

This laboratory’s interest in CD24 originated from studies on the expression of CD24 and PROM1 as markers for renal progenitor cells in the normal and injured, but not cancerous, human kidney. The progenitor properties of the PROM1 and CD24 co-expressing renal epithelial cells have been shown in multiple studies to have the capacity to participate in the regeneration of renal tubule epithelial cells [1,2,3,4,5,6,7]. It has been proposed by Romagnani and Remuzzi (2014) [8] that PROM1 expressing renal progenitor cells always co-express CD24. Primary cultures of human renal epithelial cells with retained differentiated properties of the proximal tubule (HPT) have been shown to co-express PROM1 and CD24, potentially providing a model to study the properties of renal progenitor cells. However, a study from this laboratory reported such cultures to be composed of two CD24-expressing cell types, one co-expressing PROM1 and CD24 and another expressing only CD24 [9]. Flow cytometry showed that all the cultures were composed of approximately 60% of cells expressing both PROM1 and CD24, with the remainder expressing only CD24. Through cell sorting, this allowed the isolation from the RPTEC/TERT1 cell line, two independent immortalized cell lines, one of which co-expresses both PROM1 and CD24 (HRTPT) and the other, termed HREC24T, that expresses only CD24 [9,10]. The HRTPT cells were shown to possess progenitor cell properties as evidenced by the ability to form multicellular spheroids (nephrospheres), branched tubule-like structures when grown on the surface of Matrigel and to undergo neurogenic, adipogenic, osteogenic, and tubulogenic differentiation. In contrast, the HREC24T cells demonstrated none of these features. Global gene expression analysis has been performed to determine the differential expression of genes between the two cell lines [11].

The observation that the primary cultures and immortalized cell lines all showed an approximately equal distribution of co-expressing PROM1 and CD24 cells and cells exclusively expressing CD24 suggested that the two cell types may interact with one another in a previously undefined manner. Testing this hypothesis was the first goal of the present study. A second goal was to determine whether the cultures composed of both co-expressing PROM1 and CD24 cells as well as single expressing CD24 cells could provide misleading results when used in experimental protocols. This was tested by comparing the response of these cells, when exposed to elevated concentrations of glucose, to a previous study of primary cultures of HPT cells, exposed to elevated glucose, a study investigating the effect of chronic hyperglycemia on renal epithelial cells [12]. The last goal of the study was to introduce the two new cells lines as possible models to study cancer stem cells (CSC), as defined by these two surface markers, in renal cell carcinoma. The rationale underlying their possible utility is that most CSCs are reminiscent of normal stem cells, such as the progenitor cells capable of repairing tubules of renal nephrons. An advantage of the model is the ability to examine early alterations in gene expression in agent-induced carcinogenesis before cell progression to additional stem cell lineages. 

## 2. Materials and Methods

### 2.1. Cell Culture

The RPTEC/TERT1, HRTPT, and HREC24T cells were grown using serum-free conditions as previously described by this laboratory [9,10]. For use in experimental protocols, cells were subcultured at 1:3 ratio, allowed to reach confluence and then fed with 5.5, 7.5, 11 or 16 mM glucose for 7 days followed by subculturing the cells on media containing the above elevated glucose concentrations for 3, 7 and 10 additional passages. The cells were allowed to reach confluency before subculture. Cells were fed fresh media every 3 days.

### 2.2. Exposure and Growth of HRTPT and HREC24T Cells in Conditioned Media

Serum-free media from confluent cultures of HRTPT and HREC24T cells were collected and centrifuged for 5 min at 2000 rpm. The supernatant medium was transferred into a clean conical tube and mixed in equal volume with normal growth media consisting of 1:1 mixture of Dulbecco’s modified Eagles’ medium and Ham’s F-12 growth medium supplemented with selenium (5 ng/mL), insulin (5 μg/mL), transferrin (5 μg/mL), hydrocortisone (36 ng/mL), triiodothyronine (4 pg/mL), and epidermal growth factor (10 ng/mL). HRTPT as well as HREC24T cells were fed with the mixture of conditioned/normal growth media from both cells in separate flasks, 24 h before subculture. The cells were grown in the conditioned medium until confluent and harvested for RNA isolation. 

### 2.3. RNA Isolation and Real-Time Quantitative Polymerase Chain Reaction

Total RNA was isolated using Tri Reagent (Molecular Research Center, Inc., Cincinnati, OH, USA) as described previously [12]. The measurement of mRNA expression of selected genes was assessed with real-time reverse transcription PCR (RT-PCR) and commercially available primers (Bio-Rad Laboratories, Hercules, CA, USA) as described previously [12]. The list of primers is provided in Table S1. For analysis, 1 μg of total RNA was subjected to complementary DNA (cDNA) synthesis using the iScript cDNA synthesis kit (Bio-Rad Laboratories, Hercules, CA, USA) in a total volume of 20 μL. Real-time PCR was performed utilizing the SYBR Green kit (Bio-Rad Laboratories) with 2 μL of cDNA, 0.2 μM primers in a total volume of 20 μL in an iCycler iQ real-time detection system (Bio-Rad Laboratories). Fold change was calculated using the ΔΔC_t_ method and copy number was determined using standard curves with purified and quantified PCR products for metallothionein gene expression.

### 2.4. Flow Cytometric Analysis

Confluent cultures of the renal cell carcinoma (RCC) cell lines (HTB44, HTB47 and CRL1933) were washed two times with PBS and the cells were detached using Accutase (BD Biosciences) and centrifuged at 2000 rpm at 4 °C for 5 min. The cell pellet was washed once and re-suspended in BD Pharmingen™ stain buffer (BSA). Cells were diluted to 1 × 10^6^ cells/mL with the stain buffer. For every 100 µL of cell suspension, 10 µL of fluorescein isothiocyanate (FITC) conjugated CD133 and/or phycoerythrin (PE) conjugated CD24 antibody (Miltenyi Biotec Inc., Gaithersburg, MD, USA) was added to the tubes. The cells were incubated for 30 min on ice in the dark and then washed two times with stain buffer and re-suspended in 1 mL of stain buffer. Cells were analyzed using SONY flow cytometer SP800S. The singlet population was gated as FSC vs. SSC, following which the selected population was graphed as PROM1 vs. CD24 plot. Four-quadrant data analysis was used to separate double positive vs. single positive cell populations. Based on the compensation measurements, the threshold for positive signal was set to ≤1 × 10^3^ for both markers at the *x* and *y*-axis.

### 2.5. Statistical and Bioinformatics Analysis

Statistical analysis consisted of one-way ANOVA with Tukey’s or Sidak’s multiple comparisons testing performed by GraphPad PRISM. All experiments were conducted in triplicates and the data are plotted as the mean ±SE of triplicate determinations. The STRING database contains various types of protein–protein associations. Bioinformatics analysis was performed using string-db.org [13], the query protein MT2A was searched for in *Homo sapiens*. The minimum score for the interactions was set to the highest confidence level with a value of at least 0.9, and only the first shell interactors were searched. The top 10 heavily associated proteins were selected for further analysis.

## 3. Results

### 3.1. Effect of PROM1 and CD24 Expression on the Cellular Response to Elevated Glucose Concentrations

Many studies in the literature show that the co-expression of surface markers PROM1 and CD24 identify specific progenitor cell populations within the kidney. Previous studies from this laboratory have defined the expression of PROM1 and CD24 in mortal HPT cells and the immortalized RPTEC/TERT1 cell line and both show the presence of cells co-expressing PROM1 and CD24 and another population of cells expressing only CD24. The presence of the two types of cells within the cultures suggests that studies using either the HPT or RPTEC/TERT1 are assessing the average response of the two cell populations and not their individual contributions. In addition, it also suggests that each cell type may have a role in the phenotype and genotype of the other population. Two protocols were used to test these possibilities, both of which depend on the differential expression of PROM1 and CD24 in the RPTEC/TERT1, HRTPT, and HREC24T cell lines. These three cell lines were compared to a previous study assessing the effect of elevated glucose concentrations on the HPT cells using global gene expression [12]. Validation of the results in this study confirmed that elevated glucose concentration resulted in a significant decrease in expression of 18 genes associated with the lysosome and 15 genes with mTOR. A subset of these genes was used to compare whether similar responses occurred under identical conditions for the RTTEC/TERT1, HRTPT, and HREC24T cells. 

**Figure 1:**
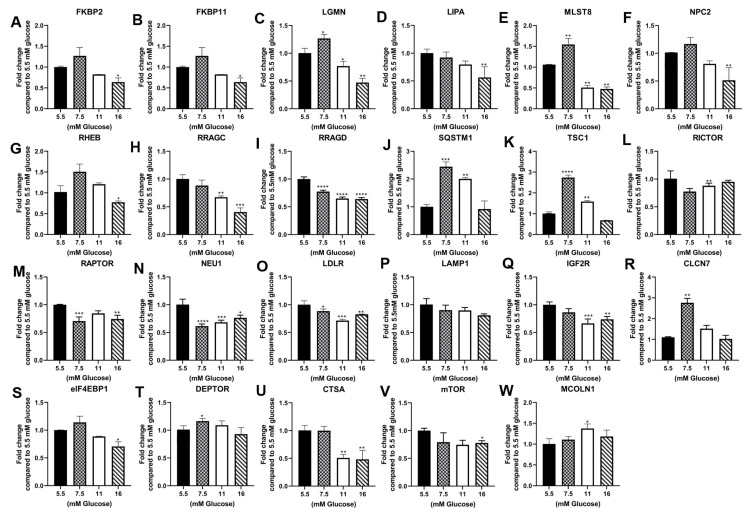
Expression of lysosomal and mTOR genes in RPTEC/TERT1 cells treated with 5.5 mM, 7.5 mM, 11 mM and 16 mM glucose for 3 passages. RT-qPCR analysis of (**A**) FKBP2; (**B**) FKBP11; (**C**) LGMN; (**D**) LIPA; (**E**) MLST8; (**F**) NPC2; (**G**) RHEB; (**H**) RRAGC; (**I**) RRAGD; (**J**) SQSTM1; (**K**) TSC1; (**L**) RIC; (**M**) RAP; (**N**) NEU1; (**O**) LDLR; (**P**) LAMP1; (**Q**) IGF2R; (**R**) CLCN7; (**S**) eIF4EBP1; (**T**) DEPTOR; (**U**) CTSA; (**V**) mTOR; (**W**) MCOLN1. ****; ***; **; * indicate significant differences in gene expression level compared to the 1.0 control 5.5 mM glucose concentration at *p*-value of ≤0.0001; ≤0.001; ≤0.01; ≤0.05, respectively.

**Figure 2:**
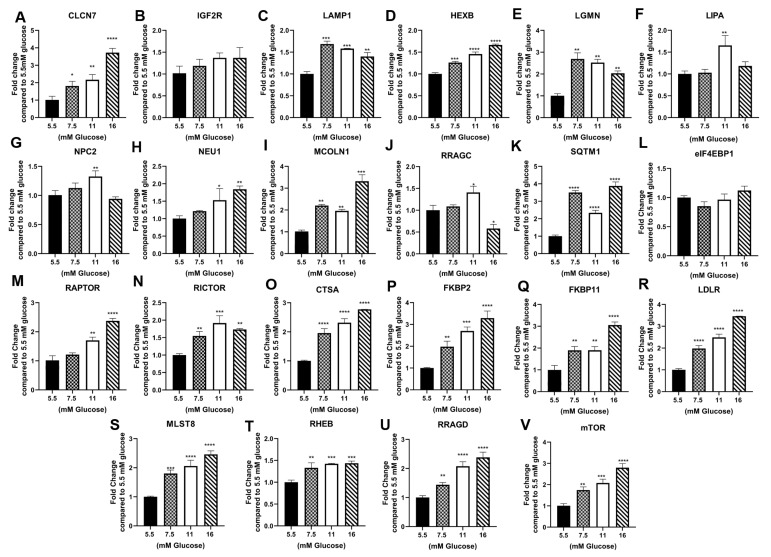
Expression of lysosomal and mTOR genes in HRTPT cells treated with 5.5 mM, 7.5 mM, 11 mM and 16 mM glucose for 10 passages. RT-qPCR analysis of (**A**) CLCN7; (**B**) IGF2R; (**C**) LAMP1; (**D**) HEXB; (**E**) LGMN; (**F**) LIPA; (**G**) NPC2; (**H**) NEU1; (**I**) MCOLN1; (**J**) RRAGC; (**K**) SQTM1; (**L**) elF4EBP1; (**M**) RAPTOR; (**N**) RICTOR; (**O**) CTSA; (**P**) FKBP2; (**Q**) FKBP11; (**R**) LDLR; (**S**) MLST8; (**T**) RHEB; (**U**) RRAGD; (**V**) mTOR. ****; ***; **; * indicate significant differences in gene expression level compared to the 1.0 control 5.5 mM glucose concentration at *p*-value of ≤0.0001; ≤0.001; ≤0.01; ≤0.05, respectively.

**Figure 3:**
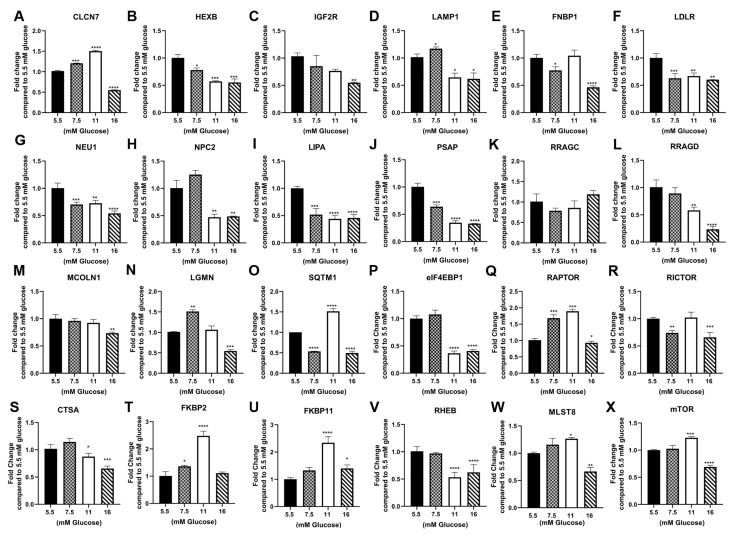
Expression of lysosomal and mTOR genes in HREC24T cells treated with 5.5 mM, 7.5 mM, 11 mM and 16 mM glucose for 7 passages. RT-qPCR analysis of (**A**) CLCN7; (**B**) HEXB; (**C**) IGF2R; (**D**) LAMP1; (**E**) FNBP1; (**F**) LDLR; (**G**) NEU1; (**H**) NPC2; (**I**) LIPA; (**J**) PSAP; (**K**) RRAGC; (**L**) RRAGD (**M**) MCOLN1; (**N**) LGMN; (**O**) SQTM1; (**P**) elF4EBP1; (**Q**) RAPTOR; (**R**) RICTOR; (**S**) CTSA; (**T**) FKBP2; (**U**) FKBP11; (**V**) RHEB; (**W**) MLST8; (**X**) mTOR. ****; ***; **; * indicate significant differences in gene expression level compared to the 1.0 control 5.5 mM glucose concentration at *p*-value of ≤0.0001; ≤0.001; ≤0.01; ≤0.05, respectively.

The analysis of the parental cell line, RPTEC/TERT1, demonstrated that the majority of the genes tested were not altered by exposure to elevated glucose concentrations at passage 3 (Table 1, Figure 1A–W). This is in contrast to the repression of these genes reported in the previously study using HPT cells [12]. A similar analysis was performed on the HRTPT cells at passage 10 and demonstrated that that elevated glucose concentrations increased the expression for most of the assessed genes, again showing a major difference with the HPT cells (Table 1, Figure 2). The expression of the selected gene set in HREC24T cells demonstrated a marked reduction in expression due to elevated glucose concentration at passage 7 (Table 1, Figure 3). The HREC24T cells response to elevated glucose was similar to that published previously for the HPT cells. Thus, the results show that cells which express CD24, but not CD133, decrease the expression of lysosomal/mTOR genes when exposed to elevated glucose. In contrast, cells which co-express the CD24 and CD133 genes react to elevated glucose by increasing the expression of the lysosomal/mTOR genes. The comparison of the two cell lines had to be performed at different passage numbers since the HREC24T cells grew slower in high glucose concentrations than that of the HRTPT cells, with the HRTPT cells completing passage 10 by the time the HREC24T cells had completed passage 7. 

### 3.2. Metabolic Crosstalk between Cells as a Function of CD24 and CD133 Expression

#### 3.2.1. MT1A, MT1E, MT1F, MT1X and MT2A Expression in HRTPT and HREC24T Exposed to Conditioned Media

**Figure 4:**
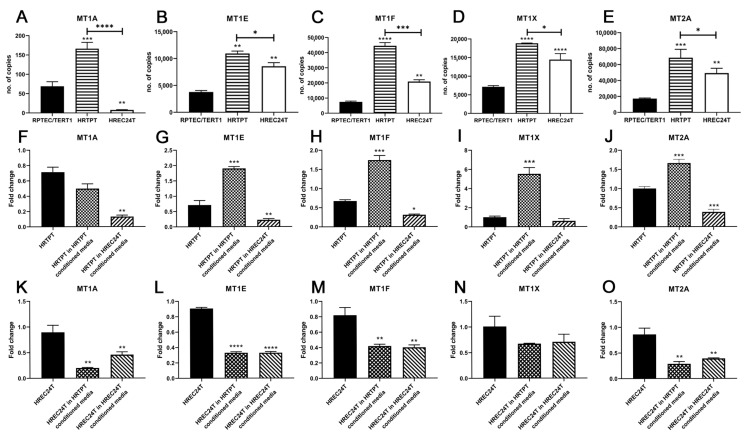
Expression of metallothionein genes in HRTPT and HREC24T cells exposed to conditioned media. RT-qPCR analysis of (**A**) MT1A, (**B**) MT1E, (**C**) MT1F, (**D**) MT1X, (**E**) MT2A in RPTEC/TERT1, HRTPT and HREC24T cells; (**F**) MT1A, (**G**) MT1E, (**H**) MT1F, (**I**) MT1X, (**J**) MT2A, in HRTPT control, HRTPT exposed to conditioned media from HRTPT, and HREC24T cells exposed to conditioned media from HREC24T; (**K**) MT1A, (**L**) MT1E, (**M**) MT1F, (**N**) MT1X, (**O**) MT2A in HREC24T control, HRTPT exposed to conditioned media from HREC24T and HREC24T cells exposed to conditioned media from HREC24T. ****; ***; **; * indicate significant differences in copy number and gene expression level compared to the control either RPTEC/TERT1 or HRTPT or HREC24T at *p*-value of ≤0.0001; ≤0.001; ≤0.01; ≤0.05, respectively.

Metallothionein (MT) expression was chosen to assess possible crosstalk between the two cell populations since its basal expression is relatively specific to the proximal tubule. The metallothionein gene family expressed in the kidney and under basal conditions confined to the proximal tubule [14]. MT expression is also found in the developing kidney with expression developing the moment differentiation is completed from the S-body [14,15,16]. Since these populations appear to have progenitor cell-like properties and since the MT expression is relatively specific for proximal tubule cells, the MT gene family was assessed in HRTPT and HREC24T cells to determine whether these genes could be used to detect cross-talk between the two cell lines. Five MT genes were chosen for analysis: MT1A, MT1E, MT1F, MT1X and MT2A. The basal expression of the five MT genes varied in a range of a low level of expression for the MT1A gene, moderate expression levels for the MT1E, MT1F and MT1X genes, and the highest level for the MT2A gene (Figure 4A–E). The comparison of MT expression between the HRTPT and HREC24T cell lines showed that basal expression of all five MT genes was elevated in HRTPT cells compared to the parental RPTEC/TERT1 cells and for four of the five MT genes (MT1A is reduced) of the HREC24T cells. When HRTPT cells and HREC24T cells were compared, the HRTPT cells expressed higher levels of all five MT genes. The basal levels of MT expression in the HRTPT and HREC24T cells were used to determine whether conditioned growth media from one cell line would alter MT gene expression in the other. For this determination, conditioned media were collected from either the HRTPT or the HREC24T cells 24 h after feeding with fresh growth media, used at a 50:50 dilution with fresh growth media, and then used to refeed confluent cultures of the two cell lines. As such, the HRTPT cell line was exposed to conditioned media from HREC24T cells and from the HRTPT cells. Similarly, the HREC24T cells were exposed to conditioned media from the HRTPT cells and from the HREC24T cells. The cells were harvested at confluency following the feeding of the cells with conditioned media. The results showed that the HRTPT cells treated with conditioned media from the HREC24T cells elicited a significant decrease in the expression of all five MT genes (Figure 4F–J). The exposure of the HREC24T cells to conditioned media from the HRTPT cells also showed a reduction in MT expression for four of the five MT genes, with the MT1X gene showing a non-significant trend for reduced expression (Figure 4K–O). As a putative control, conditioned growth media from the HRTPT cells were added back to the parent HRTPT cells. While it might be expected that this would have no effect on MT expression, the addition of conditioned media back to the originating cell line produced a significant increase for four of the five MT genes, with MT1A being unaffected by the addition (Figure 4F–J). The addition of conditioned media from HREC24T cells to the HREC24T cells resulted in a reduction in expression of four of the five MT genes, with MT1X only showing no significant reduced expression.

#### 3.2.2. MT2A-Associated Gene Expression in HRTPT and HREC24T Exposed to Conditioned Media

**Figure 5:**
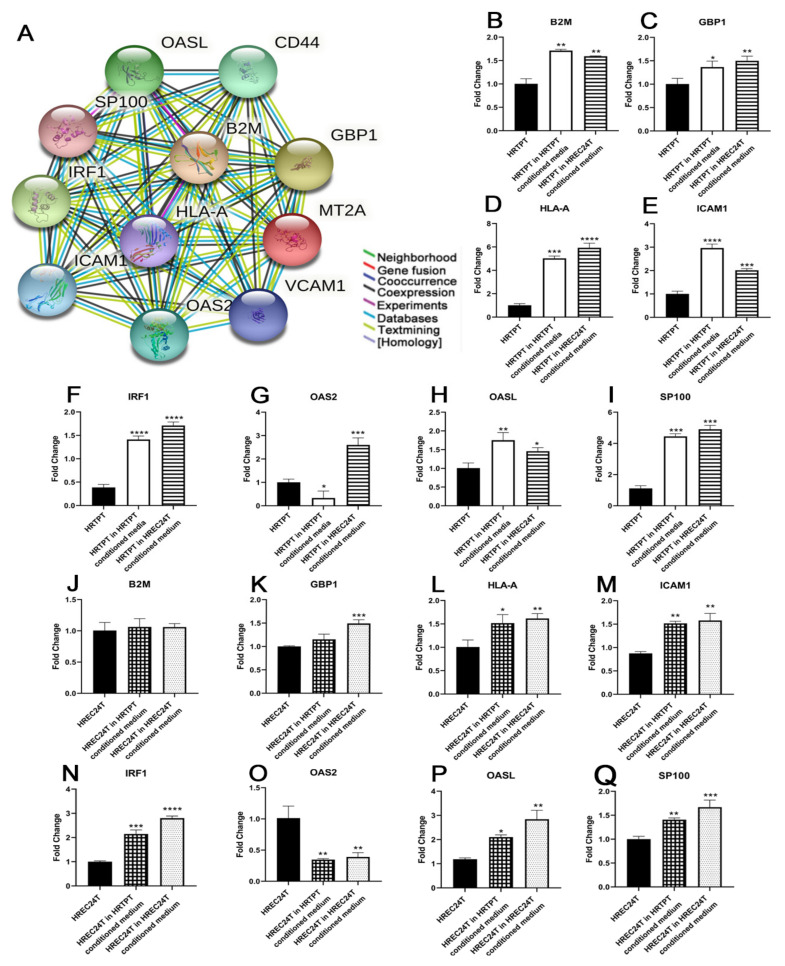
Expression of MT2A associated genes in HRTPT and HREC24T cells exposed to conditioned media. (**A**) MT2A showed numerous protein–protein associations, and the top 10 genes with highest string-db score were OAS2, OASL, HLA-A, SP100, B2M, CD44, GBP1, VCAM1, ICAM1, and IRF1. RT-qPCR analysis of (**B**) B2M; (**C**) GBP1; (**D**) HLA-A; (**E**) ICAM1; (**F**) IRF1; (**G**) OAS2; (**H**) OASL; (**I**) SP100 in HRTPT control, HRTPT exposed to conditioned media from HRTPT and HREC24T cells exposed to conditioned media from HREC24T; (**J**) B2M; (**K**) GBP1; (**L**) HLA-A; (**M**) ICAM1; (**N**) IRF1; (**O**) OAS2; (**P**) OASL; (**Q**) SP100 in HREC24T control, HRTPT exposed to conditioned media from HREC24T and HREC24T cells exposed to conditioned media from HREC24T. ****; ***; **; * indicate significant differences in gene expression level compared to the control either HRTPT or HREC24T at *p*-value of ≤0.0001; ≤0.001; ≤0.01; ≤0.05, respectively.

Several additional genes related to the MTs were assessed for their expression in HRTPT and HREC24T cells exposed to conditioned media. This was performed to determine whether the inhibitory effect of conditioned media on MT expression in HRTPT and HREC24T cells spread beyond just the MT genes. STRING was used to analyze MT-related protein networks [13], and 8 MT2A-associated genes (OAS2, GBP1, IRF1, SP100, HLA-A, B2M, ICAM1, OASL) were chosen for further study since they showed an uncomplicated relationship with MT2A (Figure 5A). These genes showed biological pathway enrichment for interferon-gamma-mediated signaling pathway, innate immune response, and regulation of cell adhesion. Conditioned growth media were also shown to alter the expression of many of these genes. HRTPT cells exposed to HREC24T conditioned media showed increased expression of all 8 MT2A-related genes (Figure 5B–I). HRTPT cells exposed to their own conditioned media showed an increase in expression of seven genes, with OAS2 showing a decrease in expression (Figure 5G). HREC24T cells exposed to HRTPT conditioned media showed an increase in expression of five genes (IRF1, SP100, HLA-A, ICAM1, OASL), a reduction for one gene (OAS2), and no change for two genes (GBP1, B2M) (Figure 5J–Q). 

The results were similar for HREC24T cells exposed to their own conditioned media, for which all the genes except OAS2 and B2M showed upregulated expression, and OAS2 showed lower expression and B2M showed no change compared to the control HREC24T. 

### 3.3. Flow Cytometric Analysis of PROM1 and CD24 Markers in the RCC Cell Lines

Three RCC cell lines were assessed for the expression of PROM1 and CD24 markers using flow cytometry. HTB47 and HTB44 showed a very low number of cells co-expressing PROM1 and CD24; all cells were negative for PROM1 alone; and 50.3% and 96.5% cells were positive for CD24 in the HTB47 and HTB44, respectively (Figure S1A,B). CRL1933 showed 21.25% of cells co-expressed PROM1 and CD24; 27.86% cells positive for CD24 alone, and 0.3% of cells positive for CD133 alone (Figure S1C). The two different populations from the CRL1933 (Figure S1C, squared regions) were sorted into 96 well plates; however, the sorted cells failed to grow even after multiple attempts. The basal mRNA expression also validated the expression of CD133 to be significantly lower than that of the CD24 in all three RCC cell lines (Figure S1D,E).

### 3.4. Expression of HNF Genes in the HRTPT and HREC24T Cells Exposed to High Glucose

The expression of mRNA for the HNF pathway was determined for the HRTPT and HREC24T cells exposed to elevated glucose for several reasons. The hepatocyte nuclear factor-regulated genes (HNF), specific for proximal tubule cells, are an integral part of clear cell and papillary RCC transcriptomes and appear to correlate with tumor progression [17,18]. The precursor of RCC is derived from the cells of the renal proximal tubule [17,18]. There is also an increased risk of RCC in patients with diabetes, providing a potential role of elevated glucose in RCC development [19]. Global gene expression of the HRTPT and HREC24T cells also identified HNF as an upstream regulatory pathway for the HREC24T cells [11]. These observations motivated an examination of the proximal tubule HNF gene in the HRTPT and HREC24T cells exposed to elevated glucose concentrations. The results of this determination showed that the HRTPT cells at P10 showed that elevated glucose concentration caused a dose-dependent increase in HNF1A (Figure S2D). Otherwise, the expression of HNF1A at PI and HNF4A and HNF4G at P1 and P10 showed no consistent difference in expression due to glucose concentration (Figure S2A–F). While several concentrations did show a significant difference in expression, the inconsistency and low levels of change with glucose concentration for each gene cast doubt on the overall significance of the observations. The HREC24T cells showed no alteration of HNF1A, 4A or 4H as a function of glucose concentration (Figure S2G–I). The HREC24T cells did show an increase in HNF1A and HNF4A expression at elevated glucose concentrations, but without evidence of a dose response (Figure S2J,K). HNF4G was unaffected by glucose concentration in the HREC24T cells (Figure S2L). 

## 4. Discussion

A previous study from this laboratory determined the effect of long-term exposure of elevated glucose concentrations to HPT cells in culture [12]. These cell cultures are isolated from human renal cortical tissue and have a limited lifespan in culture, between 5 and 10 serial passages at a 1:3 subculture ratio. It was subsequently shown that both the HPT cells and their immortalized derivative RPTEC/TERT1 were composed of two cell populations based on the expression of PROM1 and CD24 [9]. The immortalized line, RPTEC/TERT1, was used to isolate the two populations based on their differential expression of PROM1, HRTPT, where the cells co-express PROM1 and CD24 (present at 60%), and the other, HREC24T, that expresses CD24 (present at 40%) but not PROM1 [10]. These two new cell lines and their parent presented several related questions. First, did the presence of the two cell types within the parental cultures affect the results when the cells were exposed to elevated glucose concentrations? The results showed that the effect of elevated glucose concentration was markedly different between the parental culture and the two-derived cell lines. Although not a perfect correlation, the parental line showed a trend for the expression of the 18 genes chosen for analysis to be the average of the two derived cell lines, especially at the longer duration of exposure. The HRTPT cells increased expression of the 18 gene set due to glucose exposure, while the HREC24T cells decreased expression. The study delineates two new immortalize proximal tubule-like cell lines based on the expression of PROM1 and CD24. The previous study used HPT cells that showed a marked reduction in 21 lysosomal- and mTOR-related genes, which were used to compare the basal expression of these genes in the HRTPT and HREC24T cells. The HRTPT and HREC24T cells were compared using global gene expression to determine the differentially expressed genes between the two cell lines [11]. This comparison showed that only a few of the 35 lysosomal genes and 15 mTOR genes identified as being altered by elevated glucose concentrations present in either the HRPT or HREC24T cell lines. None of the 35 lysosomal genes and only one mTOR-associated gene (OGT) identified with genes differentially expressed when HRTPT cells were compared to HREC24T cells. For the HREC24T cells, the comparison showed two lysosomal genes (LGMN, PSAP) and one mTOR gene (DEPTOR) to be present in the HREC24T differentially expressed gene set. These findings suggest that both the HRTPT and HREC24T cell lines possessed a similar expression of lysosomal- and mTOR-related genes prior to glucose exposure.

The HRTPT cell line also provides an opportunity to study a proximal tubule-like renal progenitor cell; in this case, the response to elevated glucose concentration. This is important since the proximal tubule is known to be involved in type 2 diabetic-induced nephropathy and tubular repair. The cell cultures isolated from the cortex of the human kidney and placed into cell culture under serum-free growth conditions have been characterized as retaining features of the human proximal tubule [4,9,10,20]. However, approximately 60% of the cells in these cultures co-express PROM1 and CD24, cell surface markers absent in the in vivo human proximal tubule. Numerous studies have identified these renal epithelial cells co-expressing PROM1 and CD24 as having progenitor properties for tubular repair [1,4,5,6,7,21]. Studies using the HRTPT cells have shown they are capable of differentiation into multiple cell types, form tubular structures in Matrigel, and can form nephrospheres. The origin of these progenitor cells has been the subject of debate with some studies proposing a unique population of cells scattered within the kidney and others proposing the co-expressing cells simply represent de-differentiated proximal tubule cells [22]. A recent study provides strong evidence that the cells co-expressing PROM1 and CD24 in the in vivo human kidney are heterogeneous populations of dedifferentiated proximal tubule cells [22]. Regardless of in vivo origin, the HRTPT cells represent an important model for determining tubule repair in the face of nephrotoxic insult. The model is strengthened by the HREC24T cell line that does not have progenitor properties. The current study using elevated glucose provides proof-of-principle that progenitor cells respond differently compared to those without progenitor properties.

An interesting observation regarding PROM1 and CD24 expression in cultures of HPT cells is the finding that approximately 60% of the cells co-express PROM1 and CD24 with 40% expression CD24 alone. This ratio does not change significantly before senescence of mortal HPT cells and not during extended passage of the immortalized RPTEC cells [12]. It was also shown that the HRTPT cells do not lose expression of PROM1 and CD24 over extended passages and that HREC24T cells do not gain expression of PROM1 over extended passage in culture. However, stable presence of both cell populations in mortal and immortalized HPT cells provides some evidence for co-dependency between the cell populations for a presently unknown reason. The reason is not cell survival, since both populations thrive in culture when separated from one another, and it is not cell turnover, since PROM1 expression does not diminish with culture age. To gain initial evidence that soluble factors produced by the two cell types might influence gene expression of the other cell type, tests were conducted using the expression of the human metallothionein genes and conditioned growth media from the individual HRTPT and HREC24T cell lines. This analysis demonstrated that expression of the MT1A, MT1E, MT1X, MT1F and MT2A in the HRTPT and HREC24T cells was altered by exposure to conditioned media from the opposite cell type. In both instances, conditioned media placed on the same cell type also modified MT gene expression. While the explanations for this finding are subject to further investigation, it provides convincing evidence that secretion of soluble factors can influence gene expression in their own cells and those of the other cell line. The induction of MT gene expression is very well characterized, but the inhibition of expression is not well documented in the literature [23]. Indirect evidence to suggest that the HREC24T CD24 sole expressing cells and the HRTPT co-expressing PROM1 and CD24 cells might secrete factors that influence one another is suggested by observations in the developing human kidney. In the developing kidney, the PROM1 expressing cells are a subset of CD24 expressing cells which constitute the metanephric mesenchyme-derived primordial nephron [3]. During this process, the cells are in close proximity to one another, which might facilitate cross-communication. Similarly, cells expressing both PROM1 and CD24 and those expressing CD24 are only found in the adult human kidney, but their proximity to one another has not been explored following the response to renal injury. 

To the authors’ knowledge, this study is the first to show that elevated glucose concentrations elicit gene expression alterations in renal epithelial cells with progenitor properties. The in vitro evidence that renal progenitor cells have a unique response to glucose can potentially impact the mechanisms that underly renal injury and repair in type 2 diabetes, since progenitor cells must proliferate and differentiate to maintain the function of the proximal tubule during hyperglycemia. In addition, there is a distinct possibility that past studies using HPT cells as a model of the proximal tubule are compromised by the presence of a progenitor genotype and phenotype. It is likely one is studying the dedifferentiation and regeneration of renal tubules rather than fully differentiated proximal tubular cells. The previous study on the HRTPT and HREC24T cells demonstrated only 873 differentially expressed genes between the HRTPT and HREC24T cell lines, narrowing the analysis of the number of genes potentially altered by nephrotoxic agents or conditions. Renal progenitor cells are difficult to study in the intact kidney due to their sparse population and scattered locations [11]. Thus, the present cell lines may help identify factors and mechanisms involved in renal repair in the intact kidney. Envisioning a mechanism by which high glucose concentrations impact the gene expression in these cells is complicated by the fact that in progenitor cells, the metabolism may not necessarily reflect the physiology of the mature proximal tubule, but rather the dividing progenitor cell. Recent investigation into the metabolic regulation of stem cells has shown that many stem cells have metabolic signatures distinct from their mature and differentiated tissue counterparts [24]. The cellular and metabolic machinery of stem cells often reflects attenuated anabolic states with fewer organelles. The investigation of the metabolic regulation of these cells is still in its infancy. The cells in the current study probably reflect an intermediate differentiation state, most likely in a state of flux. The HRTPT and HREC24T cells offer one model system to investigate this new area of research. 

The HRTPT cells also provide a model to explore a possible role of these cells in the early development of renal cell carcinoma (RCC). The proximal tubule is believed to be the cell of origin for the development of both clear cell and papillary RCC [17,18]. In addition, there is evidence that the PROM1 and CD24 co-expressing renal progenitor cell is a heterogeneous population of dedifferentiated proximal tubule cells. The existence of the dedifferentiated proximal tubule cell following renal injury could represent an early step in the process of epithelial-to-mesenchymal transition associated with cancer development in most organ systems. There is also an association between patients with diabetes and the development of RCC, providing an additional possible linkage between the dedifferentiated proximal tubule cells response to glucose and the tubular nephropathy in diabetes. Studies show that patients with diabetes are at increased risk for the development of cancer in general [25], particularly for RCC and its progression to more serious disease [19,26,27,28]. A recent review highlights the current findings on elevated glucose, diabetes, and cancer [29]. Thus, there is substantial evidence to support studies assessing the role of elevated glucose and other injurious agents with renal progenitor cells in the early stages of multi-step carcinogenesis in RCC. An initial examination of cell lines derived from RCC for progenitor properties and the expression of HNF genes under high glucose conditions did not provide additional evidence for a role of progenitor cells and elevated glucose in the etiology of RCC.

The present study highlights that results obtained from renal epithelial cell cultures derived from the human renal cortex are influenced by the presence of both progenitor and non-progenitor renal epithelial cells. The HRTPT cell culture offers an avenue to elucidate important progenitor cell functions that may translate to enhancing renal repair in the intact kidney. 

## Figures and Tables

**Table 1:** A. Induced, Repressed or No Change in Gene Expression of RPTEC/TERT1 Exposed to Glucose at P1 and P3. B. Induced, Repressed or No Change in Gene Expression of HRTPT at P1 and P10 Exposed to Glucose. C. Induced, Repressed or No Change in Gene Expression of HREC24T at P1 and P7 Exposed to Glucose.

A. Induced, Repressed or No Change in Gene Expression of RPTEC/TERT1 Exposed to Glucose at P1 and P3						
Genes	RPTEC P1			RPTEC P3		
	Induced	Repressed	No Change	Induced	Repressed	No Change
	Average Significance (Fold Change at 16 mM)			Average Significance (Fold Change at 16 mM)		
LDLR			X		X (0.91)	
mTOR		X (0.82)			X (0.85)	
RRAGC		X (0.28)			X (0.43)	
CLCN7		X (0.24)				X
NPC2		X (0.25)			X (0.5)	
LIPA		X (0.46)			X (0.57)	
RRAGD		X (0.65)			X (0.60)	
SQSTM1			X			X
LAMP1			X			X
CTSA		X (0.43)			X (0.49)	
IGF2R		X (0.66)			X (0.65)	
LGMN		X (0.25)			X (0.46)	
MCOLN1			X			X
NEU1		X (0.53)			X (0.71)	
eIF4EBP1		X (0.26)			X (0.70)	
RAPTOR		X (0.63)			X (0.49)	
RICTOR			X			X
DEPTOR		X (0.51)				X
FKBP2			X		X (0.58)	
FKBP11		X (0.47)			X (0.56)	
TSC1		X (0.22)				X
MLST8		X (0.51)			X (0.49)	
RHEB			X		X (0.52)	
**B. Induced, Repressed or No Change in Gene Expression of HRTPT at P1 and P10 Exposed to Glucose**						
**Genes**	**HRTPT P1**			**HRTPT P10**		
	**Induced**	**Repressed**	**No Change**	**Induced**	**Repressed**	**Co Change**
	**Average Significance (Fold Change at 16 mM)**			**Average Significance (Fold Change at 16 mM)**		
LDLR			X	X (3.4)		
mTOR			X	X (2.8)		
RRAGC			X		X (0.54)	
CLCN7		X (0.59)		X (3.7)		
NPC2			X			X
LIPA			X			X
RRAGD			X	X (2.38)		
SQSTM1		X (0.70)		X (3.88)		
LAMP1			X	X (1.41)		
CTSA			X	X (2.84)		
IGF2R			X			X
LGMN			X	X (2.01)		
MCOLN1			X	X (3.27)		
NEU1		X (0.68)		X (1.73)		
eIF4EBP1			X			X
RAPTOR			X	X (2.44)		
RICTOR			X	X (1.74)		
DEPTOR			X	X (1.7)		
FKBP2			X	X (3.28)		
FKBP11			X	X (3.01)		
TSC1			X	X (2.72)		
MLST8			X	X (2.43)		
RHEB		X (0.85)		X (1.43)		
FNBP1		X (0.71)		X (2.05)		
HEXB			X	X (1.60)		
**C. Induced, Repressed or No Change in Gene Expression of HREC24T at P1 and P7 Exposed to Glucose**						
**Genes**	**HREC24T P1**			**HREC24T P7**		
	**Induced**	**Repressed**	**No Change**	**Induced**	**Repressed**	**No Change**
	**Average Significance (Fold Change at 16 mM)**			**Average Significance (Fold Change at 16 mM)**		
LDLR			X		X (0.71)	
mTOR			X		X (0.79)	
RRAGC	X (2.45)					X
CLCN7			X		X (0.55)	
NPC2		X (0.50)			X (0.48)	
LIPA			X		X (0.42)	
RRAGD			X		X (0.29)	
SQSTM1			X		X (0.49)	
LAMP1			X		X (0.73)	
CTSA			X		X (0.68)	
IGF2R			X		X (0.52)	
LGMN			X		X (0.60)	
MCOLN1			X		X (0.45)	
NEU1			X		X (0.51)	
eIF4EBP1			X		X (0.42)	
RAPTOR			X		X (0.88)	
RICTOR			X		X (0.70)	
DEPTOR			X			X
FKBP2			X			X
FKBP11			X	X (1.38)		
TSC1			X			X
MLST8			X		X (0.40)	
RHEB			X		X (0.61)	
FNBP1			X		X (0.43)	
HEXB			X		X (0.54)	

## Data Availability

Data is contained within the article and supplemental data.

## Supplementary Materials

The following supporting information can be downloaded at: https://www.mdpi.com/article/10.3390/jpm13040613/s1, Table S1: List of Primers Used for qPCR, Figure S1: Flow Cytometry Analysis of CD133 and CD24 in RCC Cells, Figure S2: Expression of HNF Genes in HRTPT and HREC24T
