# Supplementary material for: Primary and Immortalized Cultures of Human Proximal Tubule Cells Possess Both Progenitor and Non-Progenitor Cells That Can Impact Experimental Results
Source: J Pers Med. 2023 Mar 31;13(4):613. doi: 10.3390/jpm13040613 (PMC10146827; doi:10.3390/jpm13040613)
Supplement: Supplementary file 1 [file jpm-13-00613-s001.zip › jpm-1862496-supplementary.pdf]

Supplementary Materials:

Table S1. List of primers used for qPCR.

S.N.	Gene Name	Catalog Number	Company
1	CLCN7	qHsaCID0017302	BIORAD
2	NPC2	qHsaCID0008165	BIORAD
3	LIPA	QHsaCID0014810	BIORAD
4	RRAGD	qHsaCID0016675	BIORAD
5	SQSTM1	qHsaCED0045925	BIORAD
6	LAMP1	qHsaCID0005925	BIORAD
7	CTSA	qHsaCED0042210	BIORAD
8	IGF2R	qHsaCID0018295	BIORAD
9	LGMN	qHsaCED0042807	BIORAD
10	MCOLN1	qHsaCID0020976	BIORAD
11	NEU1	qHsaCED0037311	BIORAD
12	mTOR	qHsaCED0048371	BIORAD
13	eIF4EBP1	qHsaCID0005914	BIORAD
14	RAPTOR	QHsaCID0016865	BIORAD
15	RICTOR	qHsaCID0007506	BIORAD
16	DEPTOR	qHsaCID0015181	BIORAD
17	FKBP2	qHsaCED0020015	BIORAD
18	FKBP11	qHsaCED0056865	BIORAD
19	TCS1	qHsaCED0046325	BIORAD
20	MLST8	qHsaCED0046508	BIORAD
21	RHEB	qHsaCID0012643	BIORAD
22	LDLR	qHsaCID0015114	BIORAD
23	FNBP1	qHsaCID0014809	BIORAD
24	HEXB	qHsaCID0013279	BIORAD
25	HNF1A	qHsaCED0001918	BIORAD
26	HNF4G	qHsaCID0017633	BIORAD
27	HNF4A	qHsaCID0015879	BIORAD
28	MT2A		Invitrogen
29	MT1X		Invitrogen
30	MT1F		Invitrogen
31	MT1E		Invitrogen
32	MT1A		Invitrogen

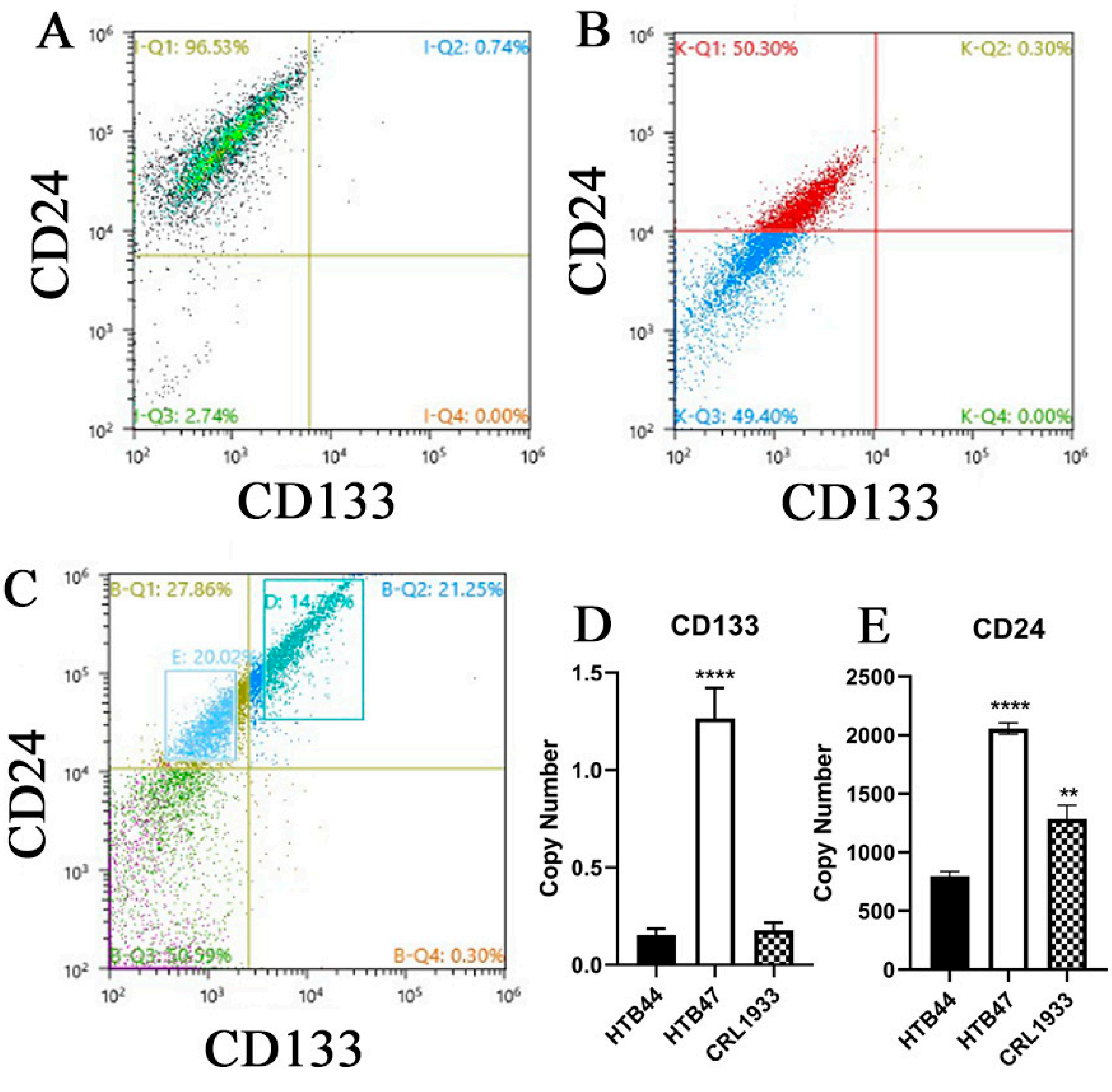

Figure S1. Flow cytometry analysis of CD133 and CD24 in RCC cells. The scatter plot XY plot is graphed as CD133 vs CD24 in (A) HTB44; (B) HTB47 and (C) CRL1933 cell lines. RT-qPCR expression of (D) CD133 and (E) CD24 in the RCC cell lines. ****; ** indicates significant differences in gene expression level compared to the control 5.5 mM glucose concentration at p-value of ≤ 0.0001; ≤ 0.01 respectively.

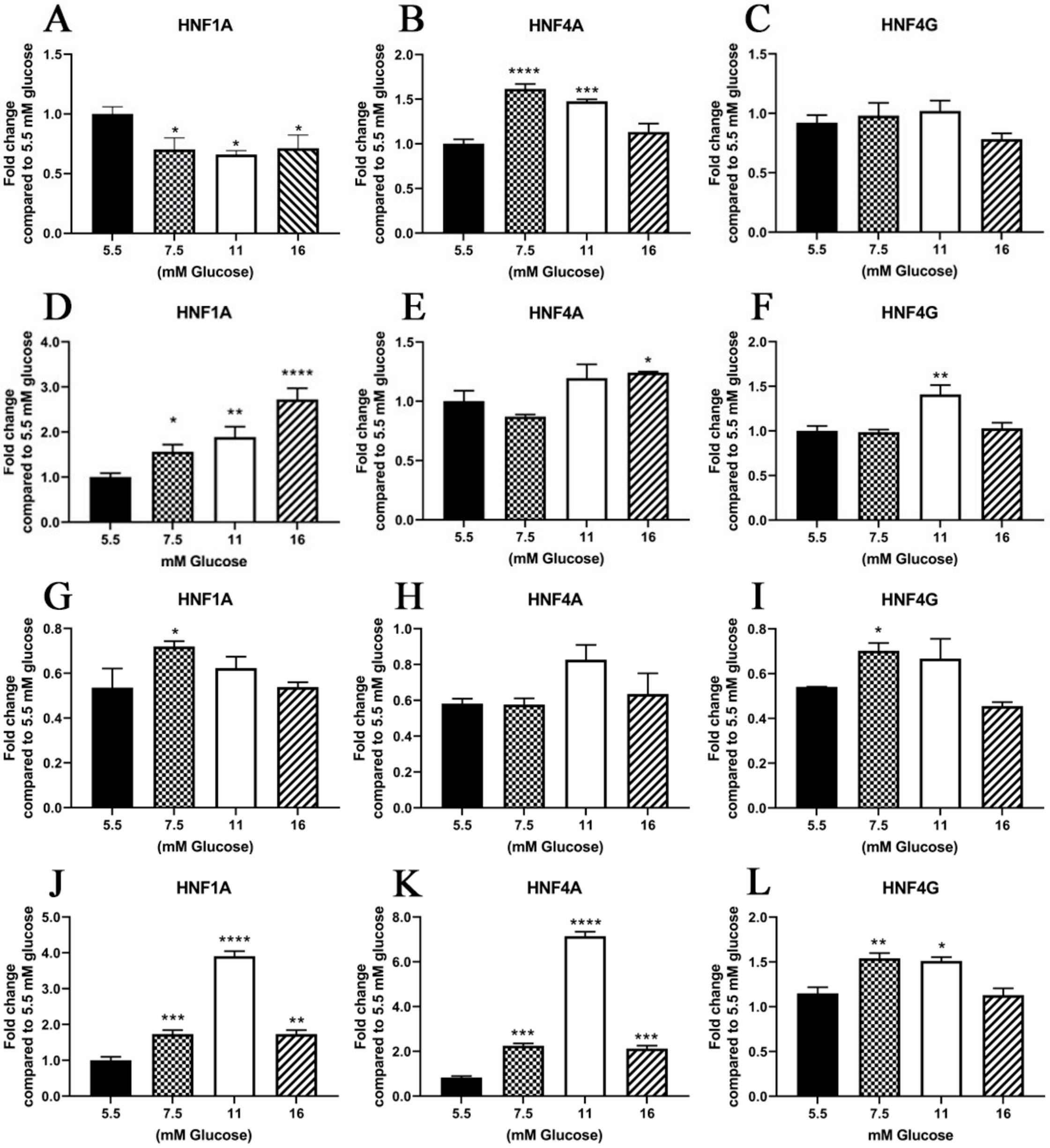

Figure S2. Expression of HNF genes in HRTPT and HREC24T cells treated with 5.5 mM, 7.5 mM, 11 mM and 16 mM glucose for 10 passages and 7 passages respectively. HRTPT cells exposed to elevated glucose for 1 and 10 passages, respectively (A) HNF1A; (B) HNF4A; (C) HNF4G and (D) HNF1A; (E) HNF4A; (F) HNF4G. HRECT24t cells exposed to elevated glucose for 1 and 7 passages, respectively (G) HNF1A; (H) HNF4A; (I) HNF4G and (J) HNF1A; (K) HNF4A; (L) HNF4G at passage seven. ****; ***; **; * indicates significant differences in gene expression level compared to the control 5.5 mM glucose concentration at p-value of ≤ 0.0001; ≤ 0.001; ≤ 0.01; ≤ 0.05 respectively.
